# Temperature and salinity, not acidification, predict near-future larval growth and larval habitat suitability of Olympia oysters in the Salish Sea

**DOI:** 10.1038/s41598-020-69568-w

**Published:** 2020-08-14

**Authors:** Jake A. Lawlor, Shawn M. Arellano

**Affiliations:** Department of Biology, Shannon Point Marine Center, Western Washington University, Anacortes, WA USA

**Keywords:** Climate-change ecology, Marine biology

## Abstract

Most invertebrates in the ocean begin their lives with planktonic larval phases that are critical for dispersal and distribution of these species. Larvae are particularly vulnerable to environmental change, so understanding interactive effects of environmental stressors on larval life is essential in predicting population persistence and vulnerability of species. Here, we use a novel experimental approach to rear larvae under interacting gradients of temperature, salinity, and ocean acidification, then model growth rate and duration of Olympia oyster larvae and predict the suitability of habitats for larval survival. We find that temperature and salinity are closely linked to larval growth and larval habitat suitability, but larvae are tolerant to acidification at this scale. We discover that present conditions in the Salish Sea are actually suboptimal for Olympia oyster larvae from populations in the region, and that larvae from these populations might actually benefit from some degree of global ocean change. Our models predict a vast decrease in mean pelagic larval duration by the year 2095, which has the potential to alter population dynamics for this species in future oceans. Additionally, we find that larval tolerance can explain large-scale biogeographic patterns for this species across its range.

## Introduction

Many marine invertebrates begin their lives as tiny planktonic larvae that drift in the water column and disperse away from their parents. For sessile species, these larval periods are especially important as they are the only times throughout life history during which organisms are capable of dispersal. As such, survival during the larval phase is critical for the persistence of populations. Larvae are highly sensitive to environmental conditions^1,2^ and the vast majority of larvae do not live to competence, so population demographics and geographic distributions of species are closely related to patterns of larval survival and metamorphosis along environmental gradients^3,4^. Thus, responses of early life-history stages to the environmental conditions in the larval habitat help to explain and predict the structures of communities in coastal oceans.

Understanding environmental influence on life-history bottlenecks is particularly important as climate variables that affect fitness are rapidly changing. Though the list of anthropogenically-influenced climate variables is broad and regionally variable, three of the most important environmental factors to consider are ocean temperature, acidification, and salinity. Broadly, temperature influences physiology of ectotherms, and thermal tolerances largely dictate distributions of marine organisms^5^; changes in ocean temperature can cause changes in developmental rate and survival that delimit range boundaries of species^6,7^. Acidification, or the shift of carbonate chemistry of a system, can affect calcification of animals with carbonate skeletons^8,9^ and, thus, will disproportionately affect many essential ecosystem engineers in marine systems such as corals, bivalves, and crabs^10^. Changes in ocean salinity affect cellular processes such as osmotic regulation and respiration in marine animals^11^, and sustained periods of low salinity can lead to mass die offs of intertidal populations^12^. Further, these stressors interact in coastal environments, and impacts of combined stressors often operate synergistically, highlighting the importance of studying stressors in combination^13–15^. By the year 2,100, climate models predict between 2 and 5 °C rise in sea surface temperatures, a pH drop of up to 0.4 pH units, and more frequent pulses of freshwater in coastal regions^16–18^. Better understanding of how these changes will influence marine species is increasingly important for conservation and resource management in this time of rapid global change.

Oysters are pertinent models for climate change studies because they are calcifying invertebrates that depend solely on the larval phase for dispersal and they have immense ecological and economic importance^19^. Here, we analyze interacting influences of temperature, salinity, and acidification on larvae of the Olympia oyster, *Ostrea lurida*. Once a major fishery on the U.S. West Coast, this species now exists at small fractions of its historical numbers due to decades of overharvesting, pollution, and habitat destruction^20^. Now, *Ostrea lurida* is a species of key regional concern on the U.S. West Coast. In Washington state, the Department of Fish and Wildlife, in collaboration with Tribal governments and conservation NGOs, has identified 19 restoration sites for the species in the Salish Sea, with the goal of repopulating selected bays with self-sustaining Olympia oyster populations^21^. Restoration efforts include out-planting of hatchery-raised seed into restoration sites, but further establishment of populations will rely on natural larval settlement. Predicting environments in which larvae can thrive will help to predict future success of populations and more precise targets for restoration efforts.

We tested the influence of environmental factors on growth rate and pelagic larval duration (PLD)—or the time between release and settlement—of *O. lurida* larvae, and on suitability of larval habitats to facilitate larval survival. In bivalves, we generally expect acidification to reduce larval growth rate and increase PLD^10,22–26^, warming to reduce PLD but have variable effects on larval growth^6,25,27^, and hyposalinity to decrease larval growth and increase PLD^28^. Slower growth rates and longer PLDs may lead to lower survival but longer dispersal potential for these larvae, depending on their behaviors^29,30^. Growth rate and PLD determine the timeframe over which larvae can access ocean currents for dispersal, thus, changes in these factors influence population connectivity as well^30,31^. Further, the maximum extent of a species’ distribution in any given spawn season can be outlined by the suitability of the larval habitat for survival because larvae are more sensitive to environmental stress than adult stages^1,2^. Certain habitat conditions may support adult oysters, but if those conditions do not support survival of larvae to competency, those habitats will fail as sources of viable larvae or as sites for new adult populations. For bivalves, we generally expect acidification^10,25,32–34^, warming^25^, and freshening^28,35^ to decrease larval habitat suitability, which may have broader implications for this species’ distribution in the future.

**Figure 1:**
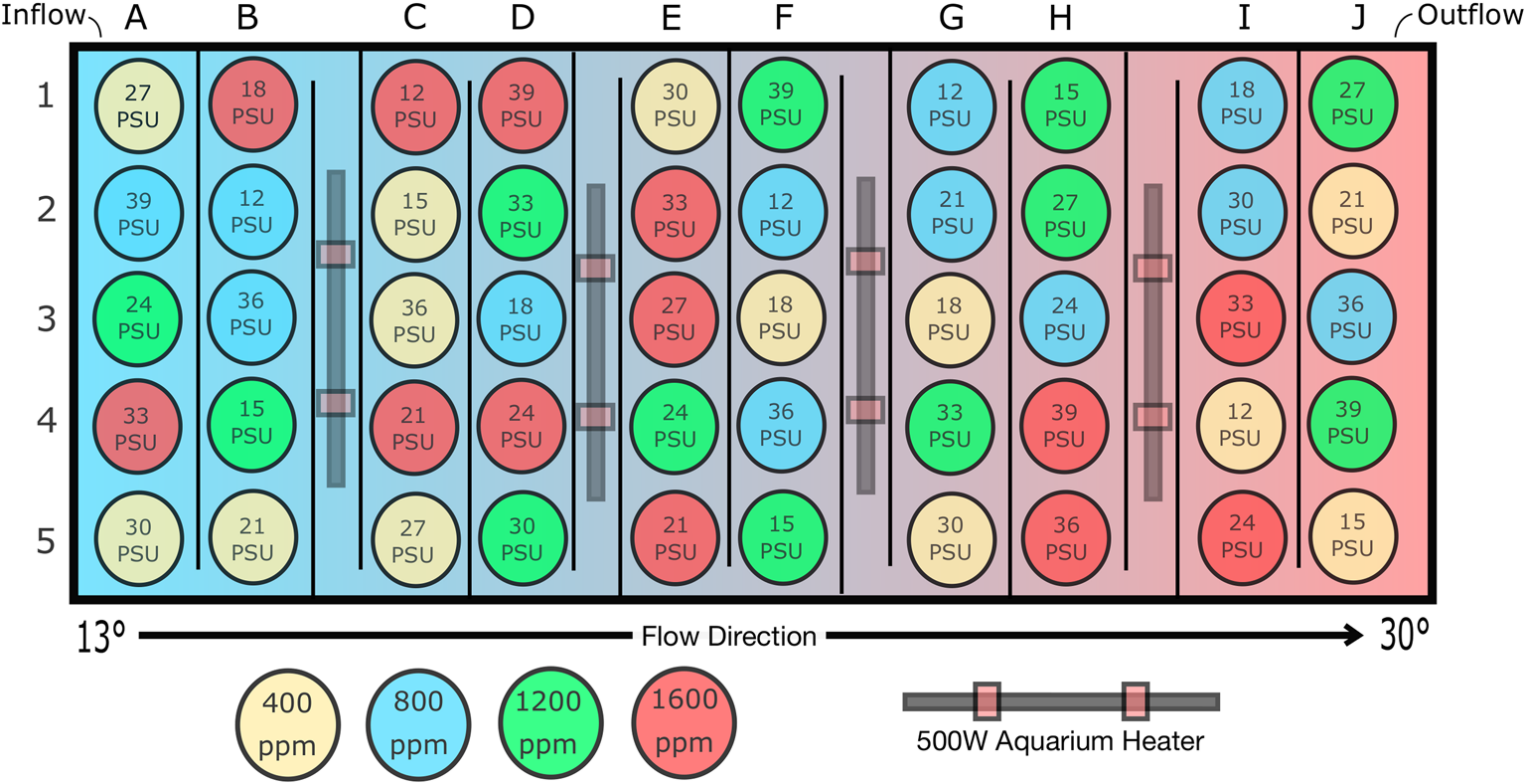
Growth experiment tank layout. The environmental gradient culturing tank houses 50 32 oz PET cups (circles) in which larvae were cultured. Each cup contains water of unique combinations of temperature, salinity, and acidification. Water enters the tank from the left side, then flows directionally across a series of weirs (black vertical lines) toward the outflow on the right. Along the way, water passes through four heating chambers each containing a 500 W digital submersible aquarium heater, raising temperature of the water before it flows into the next 10-cup chamber. This achieved a heat gradient from approximately 13°–30° through the tank (blue-red gradient). Culture cups in each group of 10 were randomly assigned an air CO_2_ concentration treatment of 400, 800, 1,200, or 1,600 ppm (denoted by circle color), with which cups were continuously bubbled throughout the experiment, and a salinity value from 12–39PSU in intervals of three (denoted by text in cup). All four CO_2_ values and ten salinity values are present within each 10-cup temperature group. Schematic manually drawn by J. Lawlor in Inkscape vector graphics software (inkscape.org).

Interactions between stressors can be complex^28,36^, and traditional multifactorial experimental designs are often limited by treatment value resolution, making it difficult to capture the full complexity of multidimensional functional response curves. Because conditions in the ocean do not occur in isolation or at discrete levels, we employ a novel experimental tank system to rear larvae in interacting gradients of environmental conditions. Using fifty unique experimental treatments of combined temperature, salinity, and acidification levels all housed in one tank, we test impacts of these variables on *O. lurida* larvae (Fig. 1). This unique design allows us to address continuous functional response patterns across environmental gradients while avoiding many issues of pseudoreplication associated with multi-factor studies^37^. Using Generalized Additive and Generalized Linear Modeling of these experimental data, we predict larval growth and larval habitat suitability under current conditions in the Salish Sea, and as environmental conditions continue to change.

## Results

### Larval rearing conditions

**Figure 2:**
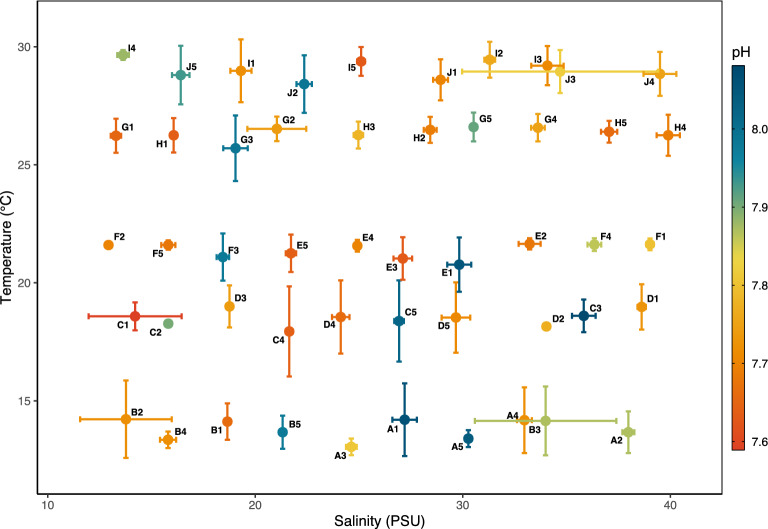
Average treatment values of culture cups in the experiment. X and Y axes represent mean salinity and temperature, color represents average pH. Error bars represent standard deviation of temperature and salinity for the duration of the cup’s inclusion in the experiment (3–17 days). Average standard deviation of salinity was ± 0.59 PSU, temperature was ± 0.83 °C, and pH was ± 0.03 pH units.

Larval rearing conditions in this experiment spanned wide ranges of each manipulated variable (13.05–29.65 °C, 12.93–39.90 PSU, and 7.59–8.08 pH) (Fig. 2, Supplementary Fig. S1–S2, Supplementary Table S1). Over the course of the experiment, treatment conditions varied due to biological factors, water changing error, and an unexpected decrease in water inflow velocity later in the experiment (Supplementary Fig. S1). The average standard deviation of conditions in our culture cups over the duration of the experiment was 0.82 °C, 0.57 PSU, and 0.038 pH units. There was much higher variation over time in pH conditions than in temperature or salinity, and thus, pH values between treatments overlapped much more than did temperature or salinity (Supplementary Fig. S1–S2). Still, most treatments remained distinct in their suites of conditions over the course of the experiment (Fig. 2).

### Larval growth

**Figure 3:**
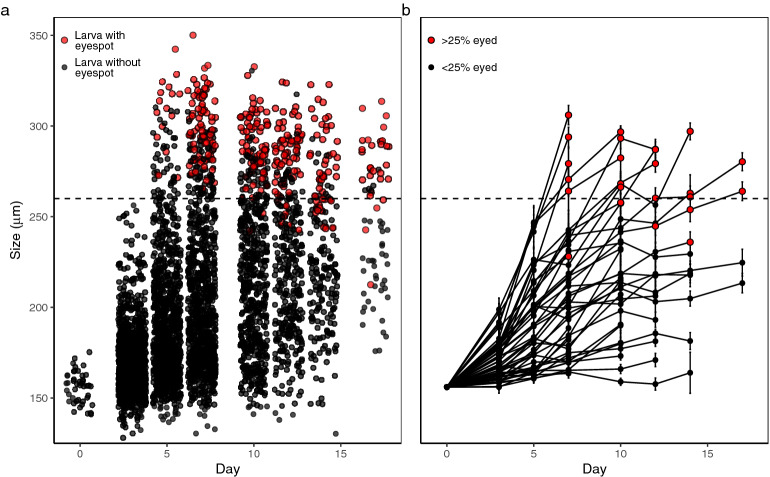
Size values through time of all larvae across experimental treatments. (**a**) Larval sizes in all treatment cups where black dots represent non-competent larvae (lacking a visible eyespot) and red dots represent competent larvae. (**b**) Average sizes with error bars ± SE for larval treatment cups. Red dots represent samples where over 25% or larvae sampled were visibly competent. In both plots, the horizontal line at 260 µm represents the size at which we considered larvae late-stage veligers.

Larvae in this experiment grew from their release sizes (135–175 µm; $\overline{x}=$ 156 ± 1.29 µm S.E.) to 350 µm and even larger as some larvae began settling in culture cups. Larvae became competent as early as day 5 in some treatments (Fig. 3a). Not all cups reached competence, so we used 260 µm as a standard beginning competence size for modeling because this was the size above which most individuals were competent and most cultures contained 25% or more competent larvae (Fig. 3b), and is consistent with size classifications from previous work in our lab^38^.

**Figure 4:**
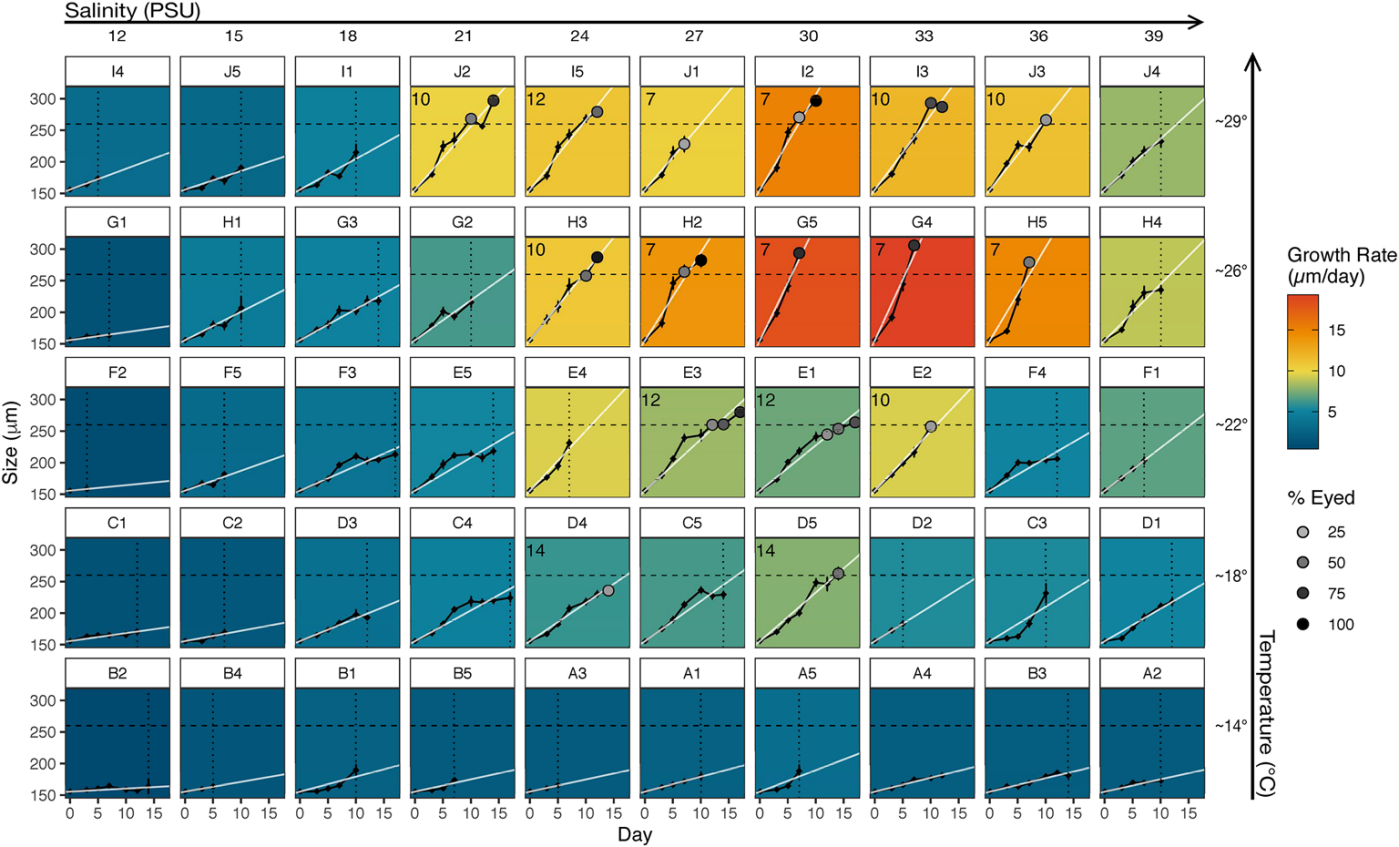
Growth of oyster larvae in 50 environmental treatments arranged by target salinity (left–right) and target temperature (bottom-top). Black lines are lengths of larval shells (n = 3–44), white lines are linear regressions, showing average growth/day. Horizontal dotted lines mark our size classification for late-stage larvae (260 µm). Filled in circles represent proportion of sampled larvae with visible eyespots at all samples > 25%. Vertical dotted lines indicate > 95% mortality. Panel color represents growth rate (µm/day). Numbers denote the day that treatments became competent (first sampling of > 25% eyed larvae).

**Figure 5:**
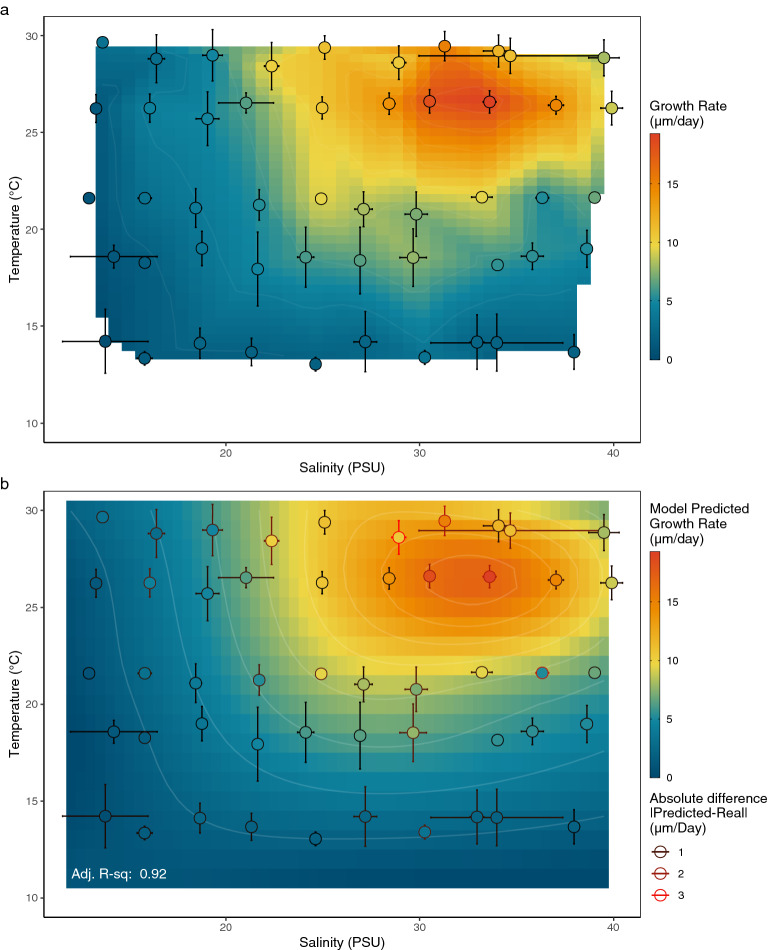
Real and predicted growth rates across environmental conditions. (**a**) Gridded Bivariate Interpolation of growth rate between experimental temperature and salinity coordinates, overlaid with real values of growth rate in experimental treatment points. (**b**) Predicted growth rate using Generalized Additive Model function at every salinity (9–39PSU) and temperature (11–30 °C) combination with experimental treatments overlaid. Color inside points represents the observed growth rate in that experimental treatment, while color of the panel represents modeled growth rate. Outline colors of points and error bars represent the absolute difference between real and predicted growth, where red outlined points are more poorly predicted by the model.

**Figure 6:**
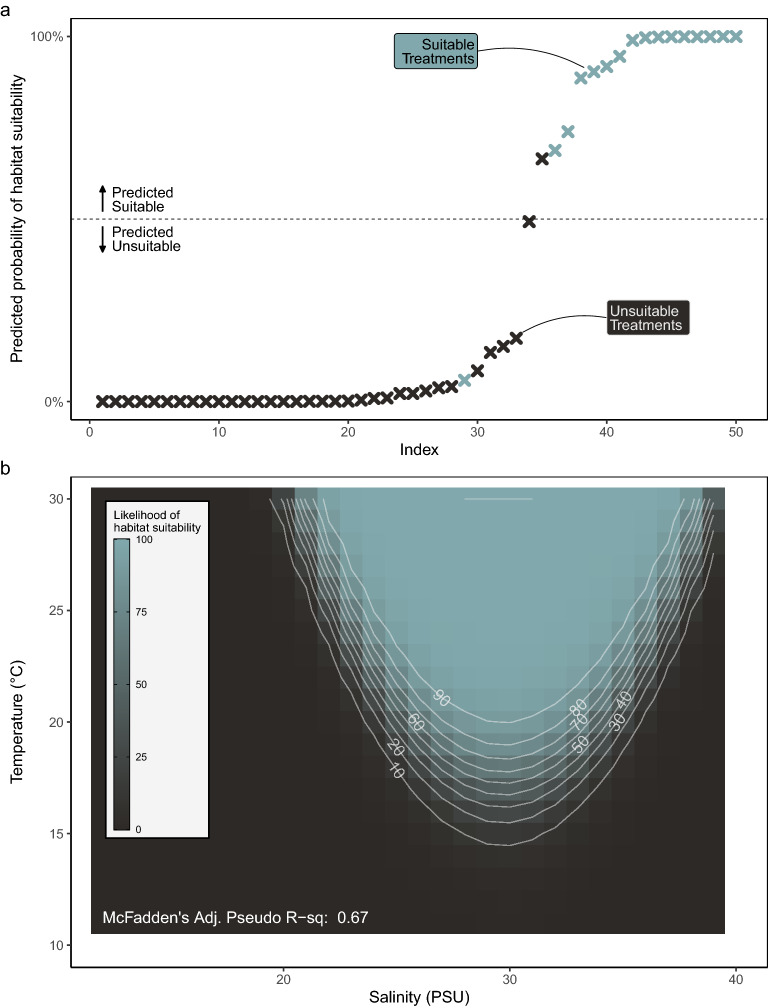
Habitat suitability likelihood of experimental treatments for Olympia oyster larvae. (**a**) Predicted habitat suitability in 50 environmental treatments. Treatments above the horizontal line are predicted as survivable. Color represents observed habitat suitability in experimental treatments. (**b**) GLM-predicted likelihood of suitability across salinity (9–39PSU) and temperature (11–30 °C). Color represents percent likelihood of 25% or more larvae growing to competency.

**Figure 7:**
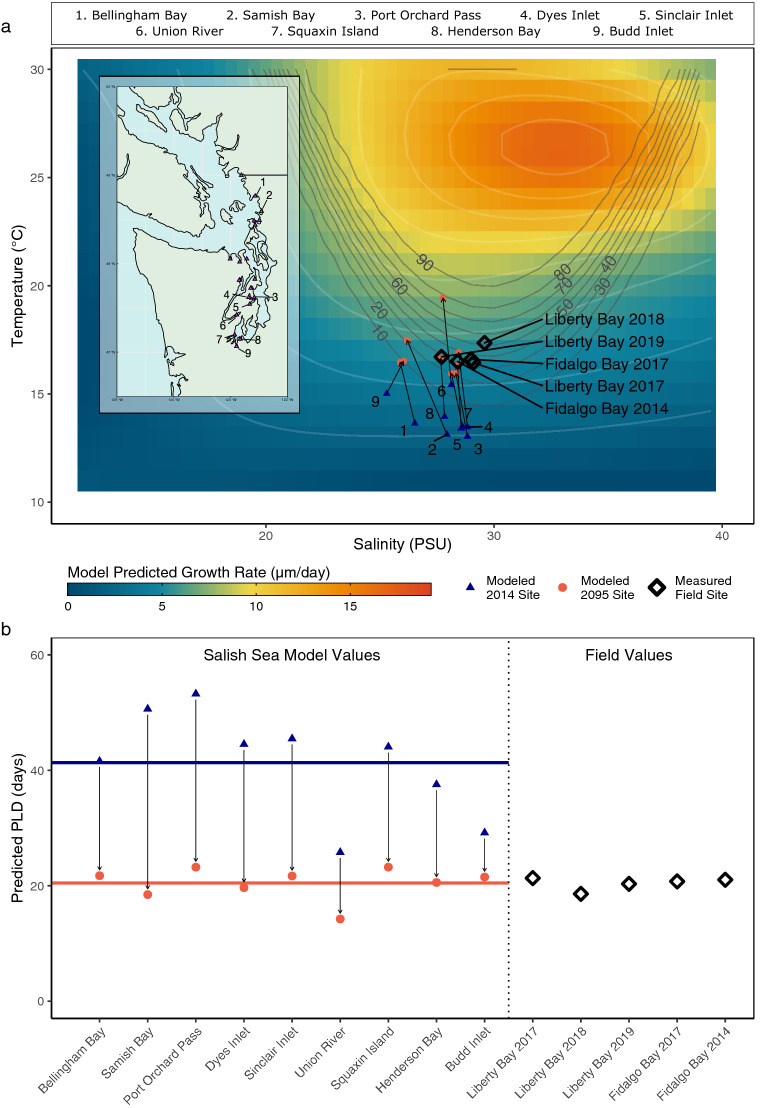
Predicted growth rates and PLDs of larvae in measured and modeled environmental conditions. (**a**) Predicted growth rate of larvae in averaged temperature and salinity values of 9 of 19 state-managed Olympia oyster restoration sites during spawning season from the Salish Sea Model in 2014 (blue triangles) and 2095 (red circles), and from field measurements (black diamonds). Numbered contour lines show habitat suitability likelihood. Inset shows site locations adapted from Blake and Bradbury^21^ created with “rnaturalearth” R package. (**b**) Predicted PLD (days from 156 to 260 µm) in each site. Horizontal lines show mean 2014 and 2095 PLD values (41.34 and 20.38 days, respectively).

Daily larval growth rates varied greatly between the 50 treatment conditions from almost no growth (0.48 µm/day) to 19.27 µm/day, with the three fastest growth rates in cups G5, G4, and H5, which all had mean temperatures at approximately 26.5 °C, mean salinities at 30, 33, and 36 PSU respectively, and at a wide range of pH values, averaging 7.91, 7.75, and 7.66 respectively (Fig. 4, Supplementary Table S1). Indeed, results from our analysis showed that temperature and salinity greatly influence daily growth rate, but larvae did not respond to acidification at this scale (Table 1a, Supplementary Fig. S3, Supplementary Table S2). No metric of acidification (total scale pH, pCO_2_, aragonite saturation) improved the fit of the model. As such, Figs. 4, 5, 6 and 7 display treatments in cartesian coordinates of temperature and salinity only, even though each treatment cup does have a unique pH value (Fig. 2, Supplementary Table S1). Model selection resulted in the final larval growth rate Generalized Additive Model (GAM):

1 $$Growth \;Rate \sim
te\left( { Temperature, \;Salinity} \right)$$

**Table 1:** Model summaries for larval growth rate and larval habitat suitability models. Model summary of Generalized Additive Model for larval growth using a full tensor product smoother (a) and Multiple Logistic Regression Model of larval habitat suitability using temperature and salinity as predictor variables (b).

(a) Generalized additive model for larval growth rate				
**Formula***: Larval Growth Rate* ~ Salinity * Temperature				
**Parametric terms**	**Value**	**SE**	**t**	***p***
(Intercept)	6.38	0.18	34.59	< 0.01
**Smooth terms**	**Estimated df**	**Ref.df**	**F**	***p***
te(Temp, Sal)	17.95	21.26	26.37	< 0.01
Adjusted R-Squared: 0.92		Deviance explained: 94.9%		
REML: 983		Scale est = 1.70		

(b) Multiple logistic regression model for larval habitat suitability				
**Formula**: *Larval Habitat Suitability* ~ *Salinity* + *Salinity*^*2*^ + *Temperature*				
**Parametric terms**	**Value**	**SE**	**z**	***p***
(Intercept)	− 122.61	46.99	− 2.61	< 0.01
Salinity	7.31	2.83	2.58	< 0.01
Salinity^2^	− 0.12	0.05	− 2.60	< 0.01
Temperature	0.81	0.33	2.48	< 0.05
McFadden’s Adjusted Pseudo R^2^: 0.67		Null deviance: 62.687 on 49 df		
Number of fisher scoring iterations: 9		Residual deviance: 12.553 on 46 df

This GAM uses temperature and salinity as interacting factors, fit with a full tensor product smoother to predict growth rate, explaining 91% of growth rate variance in experimental treatments (Table 1, Supplementary Table S2). The temperature and salinity interaction term results in a nonlinear smoothed prediction of growth rate across the two-dimensional range of model variables (Table 1, Supplementary Fig S3).

A gridded bivariate interpolation (Fig. 5a) shows the nonlinear best fit of growth rate between our experimental temperature and salinity values, while Fig. 5b shows the growth rate predicted by our growth rate GAM. Absolute difference in real vs. model-predicted growth rate peaks in cups J1, F4, and I2, in which the model overpredicts growth rate by 3.2 and 2.5 µm/day, then underpredicts by 2.4 µm/day, respectively.

### Larval habitat suitability

Of the 50 environmental treatments, 16 were considered “suitable larval habitats” with 25% or more larvae surviving to competency (Fig. 4). These 16 suitable habitat treatments had salinities centered around 30PSU, with a wider range of salinity treatments being suitable as temperatures increased. We modeled larval habitat suitability across the environmental treatment cups using a multiple logistic regression Generalized Linear Model (GLM). Our final GLM uses temperature as a linear function and salinity as a quadratic function to predict larval habitat suitability (Table 1b, Supplementary Fig. S4):

2 $$Larval\, Habitat \,Suitability \sim Salinity + Salinity^{2} + Temperature$$

Again, no metric of ocean acidification (pH, pCO_2_, or aragonite saturation) significantly predicted habitat suitability or improved the AICc of models at this scale (Supplementary Table S3).

Using this model, we correctly predicted habitat suitability in 48/50 experimental treatments, exemplifying the fit of this model to observed treatment responses (Fig. 6a). We then predicted larval habitat suitability in crossed temperatures 11–30 °C and salinities 9–39 PSU, outlining the bivariate condition thresholds that encompass potential suitable habitats for larval survival (Fig. 6b).

### Predicting impacts in the Salish Sea

Measured temperature and salinity values in two Salish Sea oyster restoration sites (Liberty Bay and Fidalgo Bay) between summers 2014 and 2019 averaged 16.44–17.36 °C, and 27.7–29.58 PSU, yielding projected average PLDs of approximately 3 weeks ($$\overline{x}$$$$=$$ 20.4 ± 1.08 days S.E.). The likelihood of suitable larval habitats in these sites ranged from 26.6% to 49.9% (Supplementary Table S4).

Temperature values in the Salish Sea Model are underestimated such that temperatures in 10 of the 19 Washington State Olympia oyster restoration sites were projected outside of the experimental bounds of our GAM (< 13 °C), and so were removed from this analysis (see methods). The remaining 9 sites averaged 13.8 °C in 2014 increasing by 1.5–4.4 °C by 2095 and averaged 27.8 PSU in 2014 changing by − 1.7 to + 0.7 PSU by 2095 (Supplementary Table S5 Using our larval growth GAM, predicted PLD decreased for larvae in every site between 2014 and 2095, ranging from a decrease of 7.7 days to a decrease of 32.1 days, averaging a 48.8% decrease in PLD in each site (Fig. 7, Supplementary Table S5).

## Discussion

Results from this experiment suggest that *Ostrea lurida* larvae are robust to these major direct impacts of climate change and that present-day conditions in the SaIish Sea are widely sub-optimal for the growth, survival, and habitat suitability of Olympia oyster larvae. Though baseline mortality was higher in this experiment than in similar studies in our lab (potentially due to poorer larval condition at the end of spawn season), relative trends in mortality still presented clear patterns. Larvae in this experiment actually performed better (faster developmental rate, increased tolerance to local habitats) in conditions representative of future environments (higher temperatures, acidified conditions, and greater freshwater influence) in the Salish Sea in the next century and beyond. Though treatment conditions were somewhat variable throughout the course of the growth experiment, results showed clear patterns of larval responses to these environmental gradients. Temperature and salinity had strong effects on both larval growth rate and larval habitat suitability, but we found no effect of any metric of acidification in this experiment.

Larvae at warmer temperatures grew faster, as is to be expected for marine larvae and most ectotherms. However, the temperature that yielded the highest growth rate in this experiment was considerably higher than we expected. Larval growth rate in this experiment peaked at around 26.5 °C, with slower growth rates at temperatures above and below this peak (Figs. 4, 5). This pattern suggests that *O. lurida* larvae from the Salish Sea can tolerate considerable warming before hotter temperatures reduce their growth rates. These trends are consistent with previous laboratory studies reviewed in Strathmann (1987) finding that *O. lurida* larvae at low temperatures (14–16 °C) show little growth and do not live past 20 days, larvae at 16–18.5 °C might grow but largely do not metamorphose^39^, and larvae in temperatures around 24° can settle in as little as 7 days^40^. Temperature also had a positive effect on larval habitat suitability, such that warmer treatments were more suitable to support larval survival, and larvae in warmer treatments could tolerate a wider range of salinities (Figs. 4, 6b). A similar pattern was identified in a model using *Crassostrea gigas* larvae^41^.The slow larval growth rates we observed at the lower temperatures common in the Salish Sea present a conundrum, as populations in the region are known to spawn at 12.5 °C and even lower in natural environments^42,43^. Even in their southern range, temperature thresholds for spawning by Olympia oysters are low (around 16 °C)^44^ given our model prediction that the likelihood of suitable larval habitat first exceeds 50% at around 17 °C and continues increasing with temperature (Fig. 6b). Better understanding of local adaptation of Olympia oyster populations, behavioral strategies of their larvae, and a finer-scale resolution of the thermal regime within the larval habitats might resolve this conundrum.

Salinity had a strong impact on larval growth and larval habitat suitability as well, but this impact was only strongly negative at levels outside the likely range of salinities in the Salish Sea in the present or near future (under ~ 21 PSU). Salinity influenced both larval growth rate and larval habitat suitability in a quadratic pattern with performance peaks at salinities around 30–33 PSU (Supplementary Fig. S3–S4), salinities which are regularly found in coastal environments where *O. lurida* live. Larval survival was low at salinities below 21 PSU, but tolerance to low salinities increased with temperature (Fig. 4). This pattern suggests a synergistic influence of these stressors such that larvae are more sensitive to suboptimal temperature and salinity when both are present but are more likely to tolerate one in absence of the other. A similar synergistic pattern has been observed regarding effects of extreme high-temperature and low-salinity events on mortality of *O. lurida* adults^45^. An increased tolerance for low salinities at higher temperature would be beneficial to Olympia oyster larvae in the Salish Sea and other estuarine systems where larvae are most likely to encounter lower salinities in freshwater lenses resting on the sea surface with relatively high temperatures.

Larvae were tolerant of ocean acidification in this experiment, including low pH, high pCO_2_, and aragonite undersaturation. This finding is consistent with other studies using this species; Waldbusser et al. (2016) found no negative response of *O. lurida* larvae to acidified conditions, in contrast to major deformation in early shell building in *Crassostrea gigas*^46^. Hettinger et al. (2013) found that *O. lurida* larvae had a slight negative response to acidification, but this response was offset by high food availability, specifically at 100,000 cells/ml *I. galbana,* the level at which we fed larvae in this experiment (see Methods)^22^. This tolerance could be due to the fact that Olympia oysters brood their larvae, as hypercapnic conditions in the brood can better prepare larvae for variable pH environments^36,47^. It is possible, however, that effects of acidification in this experiment could have simply gone undetected compared to the strong signal of the other variables. The range of acidification treatments in this experiment was 7.59–8.08 pH, or 362.12–1,205.3 pCO_2_, which might be relatively smaller than the ranges of salinity and temperature, though similar work has shown strong physiological responses to acidification within this magnitude in other bivalve species^25,48,49^. Future studies on this species should test across a wider acidification range when designing multiple factor experiments.

To better understand how these environmental responses will affect natural populations in the Salish Sea, we applied our experimental models to projected present (2014) and future (2095) oceanographic conditions in the 9 of the 19 state-managed Olympia oyster restoration sites in the Salish Sea, as predicted by the Pacific Northwest National Laboratory’s Salish Sea Model^50^. Values in this oceanographic model are biased towards deep-region values, so temperature projections for the shallow bays used in our study are considerably underestimated compared to real world values. For this reason, we used these comparisons only as identification of trends for larvae in future oceans, and not as absolute numbers. In 2095 conditions, larvae in all sites experienced faster growth rates, and larval habitats in all sites were more likely to be suitable for larval survival compared to 2014 (Fig. 7a). These growth rates yielded PLD estimates (days from 156 µm release size to 260 µm competence size) of 26–54 days in 2014, and 14–24 days in 2095, averaging a 49% decrease in PLD by the year 2095 (Fig. 7b, Supplementary Table S4). This model does not consider changes in phenology that may mediate changes in PLD for this species, and at least some populations have been shown to spawn earlier in years that warm earlier^43^ or have warmer winter conditions^51^. However, Olympia oysters can undergo several spawning events within a single reproductive season^44^, so at least some larvae are likely to encounter warm conditions in the future despite potential phenological shifts in spawning. Thus, the large decreases in PLD suggested by our growth model could have major implications for population structures and community dynamics for this species in the future.

It has been a long-standing hypothesis in larval ecology that the evolution of pelagic larval life histories developed as a mechanism for dispersal and genetic connectivity for sessile or otherwise mobility-limited marine invertebrates^52^. From this standpoint, this predicted decrease in PLD for Olympia oyster populations in the Salish Sea may further fragment populations in future oceans. However, planktonic larval stages in invertebrates might also be an evolutionary liability such that short PLD and small dispersal ranges may be beneficial to populations^53^. The presence of reproductive adults in an area implies the presence of suitable habitat for a species, so short PLD and dispersal distance would increase chances that larvae would retain access to such suitable adult habitat. While recent population genetic studies on Olympia oysters suggest high larval exchange at least at scale of the sub-basins within the Salish Sea^54,55^, the widespread historical distribution of Olympia oyster beds in the Salish Sea^21,56^ suggests that adult habitat availability is probably not limiting so high population connectivity may persist even with shorter PLDs. Moreover, shortened PLDs might decrease wastage of larvae as larvae are disproportionately vulnerable and experience exponential decreases in survivorship over time^6^. For these reasons, Olympia oyster larvae in the Salish Sea might actually benefit from some level of near-future climate change.

For a real-world representation of what *O. lurida* larvae experience in the Salish Sea, we applied our growth and larval habitat suitability models to environmental measurements taken during spawning season in multiple years at two Olympia oyster restoration sites: Liberty Bay in 2017–2019 and Fidalgo Bay in 2014 and 2017. We found that environmental conditions in these bays were fairly consistent among these five samples, and yielded average growth rates of 5.1 µm/day and PLDs of 20 days (Fig. 7, Supplementary Table S4). This estimate is slightly longer than an estimated PLD of two weeks in Fidalgo Bay in 2015 using weekly recruitment measurements on settlement plates (M. Hintz, unpublished data); the slightly shorter duration in 2015 could be explained by the limited resolution of weekly sampling, or by the presence of a marine heat wave in the Salish Sea and throughout the North American West Coast during the spawn season of that year. One caveat of these field data is that all field parameters were measured during daylight hours only, so could be slightly overestimating temperature and underestimating salinity compared to 24-h samples. Interestingly, the highest likelihood of larval habitat suitability in any of these five field samples containing Olympia oyster populations was predicted at just 49.9%, with the five-sample average being 34% (Fig. 7, Supplementary Table S4). Between the slow growth rates of larvae in experimental treatments representative of the Salish Sea and the predicted low likelihood of larval habitat suitability in local bays, it appears that Olympia oyster larvae in the Salish Sea are operating far below their environmental optima.

In the wild, Olympia oysters can be found on the North American west coast from British Columbia to Baja California. In this experiment, we observed larvae surviving to competence in water ranging from ~ 17–29 °C, and possibly higher (Figs. 4, 6b), with larvae exhibiting faster growth rates and shorter PLDs as temperatures increased (Fig. 5). At the North end of their biogeographic range, shallow bays verge near the low end of temperature tolerance limit for Olympia oyster larvae (Supplementary Table S4), while sea surface temperatures can reach the high-twenties or more during summer months near the southern end of their range (Supplementary Fig. S5). With the caveat that populations likely show some degree of local thermal adaptation, our results suggest that Olympia oysters in Washington and British Columbia are likely bound to shallow bays where temperatures are warmer, and populations in these high-latitude regions might represent the environment-driven range edge for the species.

The synergistic impacts of low temperatures and salinities on larval growth and larval habitat suitability that were evident in our experiments (Fig. 4, 5b, 6b) may impact populations of Olympia oysters differently across their range. The Salish Sea consists largely of “suboptimal” temperature environments for larvae of this species, so larvae here may be particularly sensitive to salinity stress compared to larvae from populations in warmer regions toward the southern end of their range. Together, the effects of temperature and salinity suggest that Olympia oyster larvae on the south end of their range might experience higher survival in the plankton (both due to reduced risk of mortality from physiological stress and reduced risk of predation because of shorter PLDs), and increased access to suitable larval habitats. Indeed, a coast-wide quantitative biogeographic survey of 24 historical Olympia oyster sites from Alaska to Baja showed that sites in the north end of the range more often lacked intertidal populations of Olympia oysters, while all sites in southern California supported them^57^. For these reasons, we hypothesize that Olympia oysters might exhibit a population stability gradient across latitude, as has been shown for populations of *Crassostrea virginica* on the East Coast, partially attributed to similar larval dynamics^27,58,59^. Further studies of environmental responses in later life history bottlenecks could more thoroughly test this hypothesis.

Finally, *O. lurida’s* tolerance to acidification stress is closely linked to its evolutionary history across its range. The North American West Coast is characterized by hot spots of seasonal upwelling and severe local acidification, experiencing “some of the lowest and most variable pH environments in the surface ocean” during summer months, coinciding with spawning season for Olympia oysters^60^. The tolerance to acidification by Olympia oyster larvae may reflect the species’ evolution under these low-pH conditions, as the only oyster species native to this region. Recent research even shows positive carry-over effects for Olympia oyster offspring whose parents were exposed to elevated pCO_2_ conditions^51^. Their tolerance to acidification contrasts with larval responses of other oyster species, such as *C. virginica* and *C. gigas,* which have both exhibited negative responses to acidification within the range tested in this experiment^48,49^. *C. virginica* and *C. gigas* are native to regions without such extreme pH environments, highlighting the links between species distribution, environmental tolerance, and evolutionary history for benthic and sessile marine species.

Overall, results from this experiment show clear patterns of environmental optima for Olympia oyster larvae that can be used to plan for restoration and climate change mitigation. Larvae grew faster and were more likely to tolerate habitats in warmer conditions with salinities around 30 PSU, with no effects of acidification at this scale. These results can be used to target Washington State restoration efforts to sites with conditions in which larvae will be most successful. Our finding that current conditions in the Salish Sea seem to be largely suboptimal for larvae of this species supports the suggestion of Polson and Zacherl (2009) that increased restoration efforts further south in the Olympia oyster range should be promoted as well, as these areas may have great potential for the species^57^. Though this study is limited to the larval stage only and does not consider environmental bottlenecks of settlement or metamorphosis, when focusing on the larval stage alone, our results are in agreement with recent literature that the outlook in near-future climate change scenarios is generally positive for this species^46,51^.The climate stress tolerance we find in this experiment supports this species as an optimal candidate for restoration instead of others that may be less resilient in increasingly acidifying environments on the North American West Coast. Finally, this experimental method and multi-factor gradient tank design should be used in future studies with even wider ranges of environmental variables to find tolerance thresholds, allow insight into natural history of species, and better prepare for marine resource management in the Anthropocene.

## Methods

### Experimental design

We designed a custom heat-gradient culturing tank allowing for larval culturing in 50 unique combinations of temperature, salinity, and pCO_2_. The tank featured directional flow, guaranteed by the placement of a series of weirs directing water through five heating chambers, where 500 W digital submersible aquarium heaters raised temperature of the seawater before moving across the next weir (Fig. 1). These five stable heat levels ranged from approximately 13 °C at the inflow to 28 °C on exit. Within each heat level, ten 32-oz polyethylene SOLO brand cups filled with 800 ml treatment water were randomly assigned one of ten salinity values (12–39, in intervals of 3). Each cup was bubbled with one of four pCO_2_ treatments (400, 800, 1,200, and 1,600 ppm), with temperature- and salinity-driven differences in solubility ensuring a gradient of achieved carbonate chemistry conditions between treatments. Each salinity and pCO_2_ level was present in each 10-cup temperature group. Salinities were achieved by manually mixing preequilibrated 0.35 µm filtered seawater (FSW) with concentrated brine (FSW enhanced with Marine Mix instant ocean salt) or simulated river water (deionized water enriched with sodium bicarbonate to 600µeq/L^61^ alkalinity). Acidification was achieved by individually bubbling CO_2_ controlled air into treatment cultures using an air compressor, CO_2_ scrubber, and eight mass flow controllers mixing pure CO_2_ with CO_2_-free air for treatment conditions (system described in a 2017 technical note^62^). The non-uniform multifactorial spread of this design allowed us to analyze predictor variables as continuous gradients and interpolate responses between treatment values, rather than conduct treatment level comparisons. Because all culture cups were housed in one tank, we avoided many random effects that we could have faced by splitting treatments into a limited number of incubators. It is important to note that some pseudoreplication issues persisted in this design through shared pCO_2_ tubes and row alignment within the culturing tank. Still, we designed this tank to simulate samples from 50 independent field sites, so we measured and analyzed them each individually.

### Spawning and larval rearing

All larvae for this experiment were provided by the Puget Sound Restoration Fund oyster hatchery in Port Orchard, Washington. Broodstock were collected from Mud Bay, Washington, and used in one hatchery spawn season. Broodstock from separate spawning groups were consolidated into one tank and held together for one week before larval collection. Larvae were released and collected on May 3, 2018, concentrated on a moist Nitex mesh, and shipped on ice overnight to Shannon Point Marine Center in Anacortes, Washington. Larvae were distributed into treatment cups at a target density of 2 larvae/ml on May 4, 2018. Each Monday, Wednesday, and Friday, larvae were poured from culture cups onto 100 µm Nitex screens for full water changes with preequilibrated treatment water then fed a diet of *Isochrysis galbana* at 100,000 cells/ml. We chose to feed at this level as it has been identified as optimal for larval oyster cultures^63^. Because this experiment was testing impact of specific climate stressors on larval growth, we did not want food limitation to be a confounding variable. This level is also reasonably representative of food availability in the field. McIntyre et al.^38^ measured chlorophyll-a in Fidalgo Bay (one field example in this study) averaging 19 ± 8.1 µg/L^38^. Using the estimation of 100,000 cell/ml ~ 10 µg/L Chl-a^22^, even the lower end of these field chlorophyll-a measurements corroborate our choice of feeding level as well within reason for larvae to experience in the natural environment. Thus, we assume food is not a limiting factor in larval growth in this study, nor in our extrapolations to field scenarios. While the environmental conditions manipulated in our treatment cups could potentially affect the nutritional content of algae, we chose to use *I. galbana* as food due to its common utilization in environmental stress studies^22,25,28,36,64^. Additionally, because we fed each culture following water changes three times per week from control condition algae cultures, larvae should have had the opportunity to consume algae before environmental conditions significantly affected its nutrient content. During the time larvae were condensed on screens, samples were collected to measure growth and assess mortality (see next section).

### Data collection

Three times per week, water chemistry, and larval size, developmental stage, and mortality were assessed in each culture cup. A previous experiment in our laboratory comparing carbonate chemistry sampled every 48 h from CO_2_-bubbled cultures at similar concentrations to ours with (1) seawater only, (2) *Isochrysis galbana* only, (3) Olympia oyster larvae only, and (4) both algae and larvae showed little variation in water chemistry between these four treatments over this time frame^65^. We used an Orion Star A329 multimeter to measure water temperature and salinity prior to water changes, and filtered water samples into 20 ml scintillation vials that we fixed with 20 µl mercuric chloride for later pH and DIC analysis. Fixed samples were later measured for pH using an Ocean optics S-UV–VIS flame spectrophotometer measured in a 5 cm jacketed cuvette for a baseline spectrum, then again after addition of 20 µl m-cresol dye^66^. DIC was measured with an Apollo SciTech AS-C3 DIC Analyzer, calibrated to a standard curve built from varying volumes of certified reference material (CRM, Batch 149, Dickson, Scripps Institute of Oceanography). We used measured pH and DIC values to calculate pCO_2_, pH (total scale) and aragonite saturation states (Ω) in each culture cup for each sampling event using CO2SYS^67^ with K1 and K2 equilibrium constants^68^.

To assess mortality, competence, and larval morphology, we sampled aliquots of condensed cultures until we had at least 20 living larvae on a Sedgewick rafter counting slide. In some sampling events, cups contained fewer than 20 live larvae, so we sampled the maximum number of larvae possible. All dead larvae in the aliquots were counted for mortality ratios, and then discarded. The remaining living larvae (~ 20) on the slide were fixed in 4% formaldehyde solution for 24 h, then stored in 70% ethanol in a − 80 °C freezer. Fixed samples were later photographed on a Leica M125 Stereoscope, measured for shell length using ImageJ software, and identified to developmental stage by the presence or absence of a visible eye spot. Culturing continued until culture cups reached 70% competence, 95% mortality, or the end of the larval supply, with treatment cups lasting up to 17 days post larval release.

### Analysis

We analyzed larval growth by plotting the average lengths of larval samples (n = 2–44) through time in each culture cup, starting from a common baseline sample (day 0). We ran linear regressions of larval length over time for each cup with a fixed intercept equal to average starting size (156 µm), establishing daily growth rate in each treatment culture. We suspected levels of temperature and salinity in our experiment would span the range of physiological tolerances of larvae, resulting in quadratic or otherwise nonlinear relationships. Because of this, we chose to model growth rates with Generalized Additive Models (GAM) from R package ‘mgcv,’ allowing for smoothed nonlinear relationships for individual variables. We also anticipated possible interactions between variables, so we selected GAM models from every possible combination of additive and interacting variables using temperature, salinity, and three measures of carbonate chemistry, pH, pCO_2_, and aragonite saturation (Supplementary Table S2). Values of pH and pCO_2_ were highly correlated (− 0.915), as were pH and aragonite saturation (0.567), and salinity and aragonite saturation (0.768) (Supplementary Fig. S6), so these variables were never included in models together. Aragonite saturation was not highly correlated with pCO_2_, but since both were calculated in CO2SYS from measured pH and DIC, these were not included in models together either. While we measured DIC to calculate other variables, we did not use it as a predictive factor in our models because pCO_2_, and aragonite saturation are the metrics of experimental ocean acidification that are most likely to impact larval oysters^69,70^, and pH is the most commonly studied seawater chemistry parameter^15,70^. However, our salinity treatments were created by diluting seawater with simulated river water, so DIC was correlated with salinity (0.97) in our experimental treatments. Considering this, it is possible that some salinity effects are due to DIC instead. All potential GAMs used thin plate regression spline smoothers for individual variables, and full tensor product smoothers for interacting variables. Models were compared using Akiake’s Information Criterion corrected for small sample size (AICc from R package “MuMIn”), Bayesian Information Criterion (BIC), and Adjusted R^2^ values to select the most parsimonious model (Supplementary Table S2).

To assess suitability of our experimental treatment conditions as larval habitat, we considered cups in which 25% or more of sampled larvae reached the late pediveliger stage (displaying visible eyespots) as “suitable” treatments for larval survival, or suitable larval habitats. Presence of pediveligers suggests larvae are competent to settle, and therefore are able to live through larval life in the given treatment. We chose this 25% threshold to filter out any potential false positives from cross contamination of eyed larvae from pipettes or sieves during sampling. We used the treatment values of temperature, salinity, pH, pCO_2_, and aragonite saturation state as continuous predictors, again, excluding highly correlated variables and multiple calculated carbonate chemistry variables, to create a multiple logistic regression model for habitat suitability of treatments. We used AICc, BIC, and pseudo R^2^ comparisons to select the best-fit model for these data (Supplementary Table S3).

### Predicting impacts in the Salish Sea

Having established functional responses of larval growth rate and larval habitat suitability to environmental variables, we used projected present-day and future conditions in the region to estimate in situ changes in larval growth and PLD in future Salish Sea scenarios. Then, we used measured values of environmental parameters from two specific bays where Olympia oyster populations are found to analyze larval growth and larval habitat suitability in present-day conditions in the context of the broader environmental tolerance range of the species.

To analyze near-future changes in the larval phase, we used data from the Pacific Northwest National Laboratory’s Salish Sea Model^50,71^, a hydrodynamic and water quality model of the Salish Sea, including a baseline condition in the year 2014, and a future projected oceanographic condition for the year 2095 modeled under an RCP 8.5 high CO_2_ emissions scenario. Each model includes roughly 16,000 nodes throughout the larger Salish Sea region with values at 10 sigma layers between surface and bottom at each node. We first selected nodes in the regions of each of the 19 restoration sites in Washington State that were between 0 and 20 m deep (n = 8–46 per site). Then, we averaged hourly values for relevant water quality parameters at each site between July 5—August 23 during each year. Because this model utilizes a smoothing factor for the entire Salish Sea, environmental parameters in shallow areas are biased toward deeper water values in this model; as such, environmental values in restoration site bays are skewed, with temperature being especially underestimated. Since GAM models should not be extrapolated past the limits of experimental treatments, we removed sites with average temperatures below 13°, eliminating 10 of the 19 state restoration sites. Because these 2014 and 2095 values are underestimated, we use these data as an indication of trends between 2014 and 2095, but not as absolute numbers. Using our predictive growth rate model, we predicted larval duration from release size of 156 µm to competence size of 260 µm in nine of Washington State’s active Olympia oyster restoration sites in the year 2014 and 2095. Though published estimates of competence size for Olympia oysters vary^72–74^, we chose 260 µm as the a beginning competence size as this was the size at which we began seeing frequent eye spots in our experimental cultures (Fig. 3) and is consistent with previous stage classifications from our lab^38^. We did not use these data to predict larval habitat suitability of the sites, as the low temperature values put all points in 2014 outside of the larval habitat suitability logistic regression curve.

To examine larval tolerance to conditions in the natural environment, we focused on two particular state-operated restoration sites in the Salish Sea that we know contain breeding adults of the species: Fidalgo Bay and Liberty Bay, Washington^21,35^. We used environmental data in Fidalgo Bay summarized from McIntyre et al.^38^ and Cordoba and Arellano (unpublished data); both studies measured water quality variables throughout the water column in sampling efforts during mid-July of two separate summers. We obtained data from Liberty Bay from the Western Washington University SEA Discovery Center’s long-term monitoring project, which measured water quality parameters from surface to depth twice weekly from 2017–2019, and averaged values from July 5 to Aug 23 (estimated peak larval season)^44^ during each year. With these two samples from Fidalgo Bay and three from Liberty Bay, we project PLD and suitability of habitat for Olympia oyster larvae in realistic conditions in the Salish Sea.

## Supplementary information

Supplementary information.
