# Supplementary material for: Temperature and salinity, not acidification, predict near-future larval growth and larval habitat suitability of Olympia oysters in the Salish Sea
Source: Sci Rep. 2020 Aug 14;10:13787. doi: 10.1038/s41598-020-69568-w (PMC7429507; doi:10.1038/s41598-020-69568-w)
Supplement: Supplementary file 1 — Supplementary information. [file 41598_2020_69568_MOESM1_ESM.pdf]

Temperature and salinity, not acidification, predict near-future larval growth and larval habitat suitability of Olympia oysters in the Salish Sea

Supplementary Materials

Jake Lawlor*

Department of Biology, Shannon Point Marine Center, Western Washington University, Anacortes, WA, USA,

jakealawlor@gmail.com

Shawn Arellano

Department of Biology, Shannon Point Marine Center, Western Washington University, Anacortes, WA, USA,

shawn.arellano@wwu.edu

Supplementary Figures and Tables

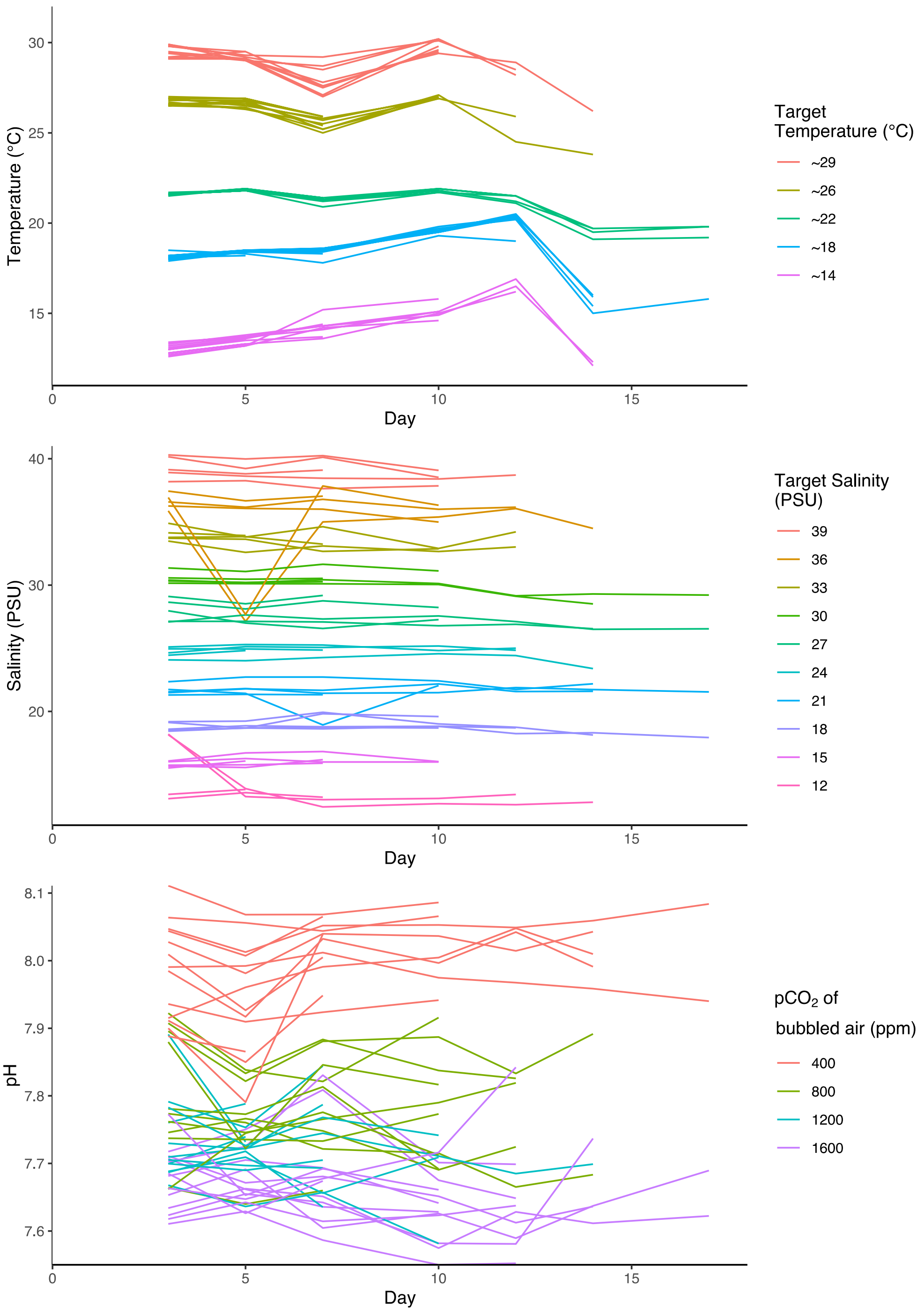

Supplementary Figure S1:Treatment cup variable stability over time from May 7-21, 2018. Each line represents one cup, colored by target treatment.

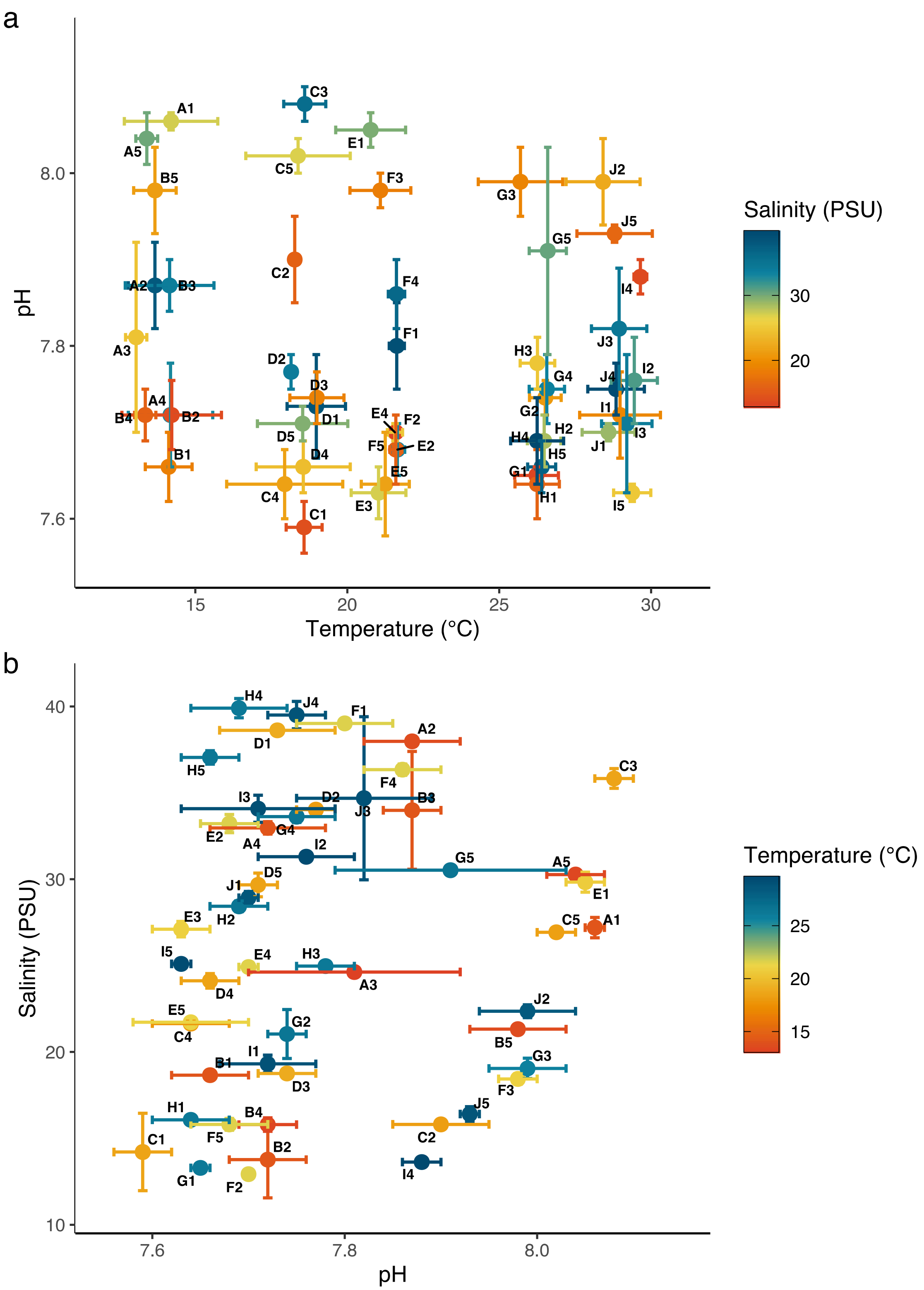

Supplementary Figure S2: Average treatment values of culture cups in the experiment organized by temperature and pH (a), and pH and salinity (b) as addendum to Fig 2, arranged by salinity and temperature. Error bars represent the standard deviation of axis variables for the duration of the cup's inclusion in the experiment (3-17 days).

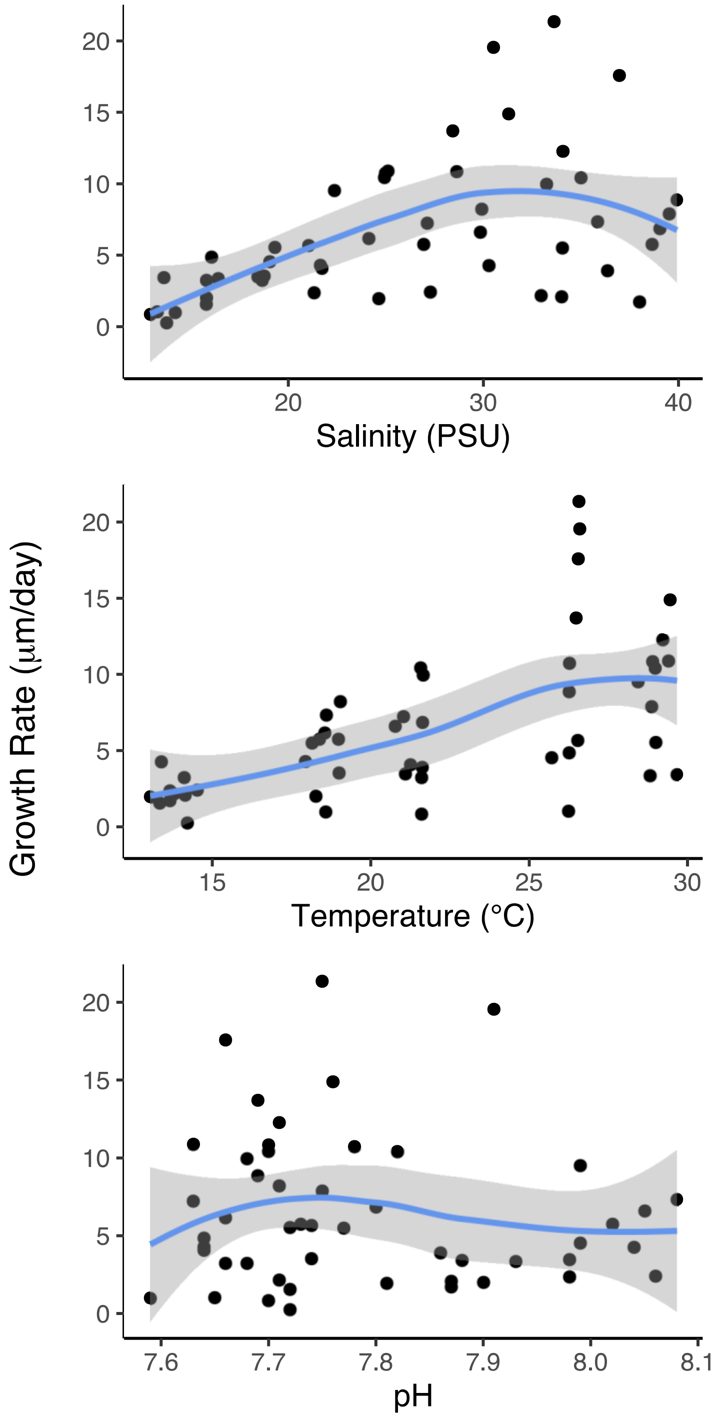

Supplementary Figure S3: Experimental growth rate distribution by variable with loess curve fits.

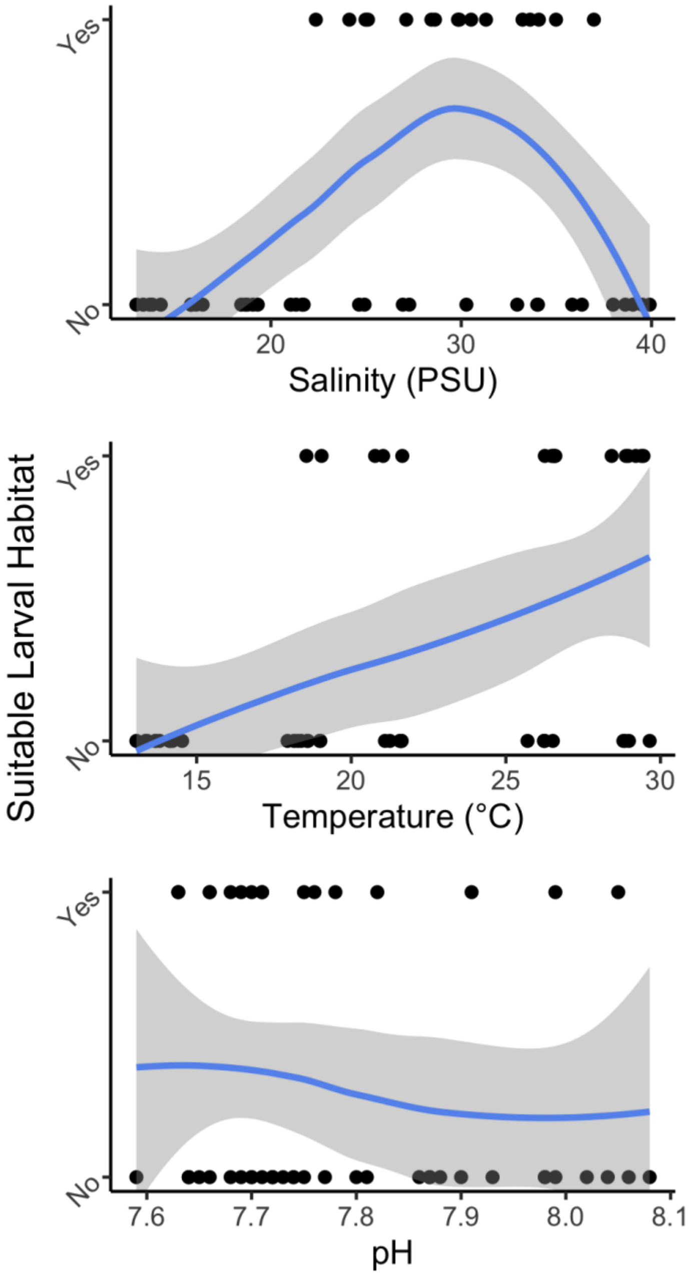

Supplementary Figure S4: Experimental larval habitat suitability by variable with loess curve fits.

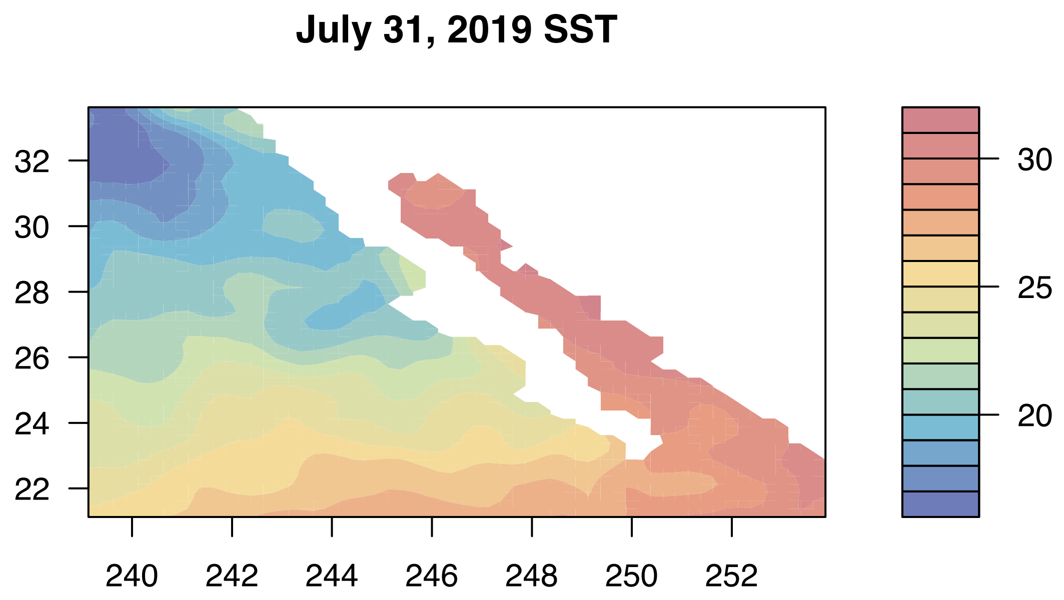

Supplementary Figure S5: Average sea surface temperature (°C) around Baja California on July 30, 2019. Satellite data acquired from NOAA High Resolution SST, NOAA/OAR/ESRL PSD, Boulder, Colorado, USA.

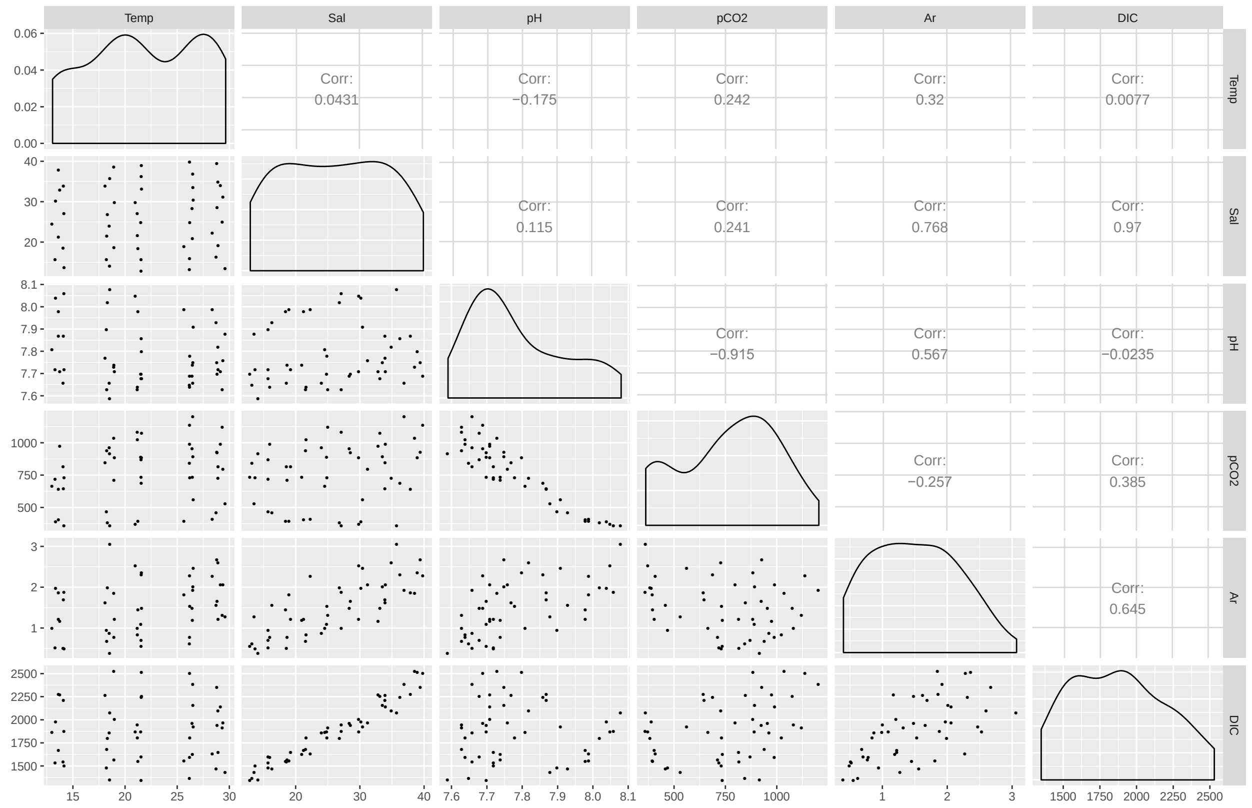

Supplementary Figure S6: Correlation matrix of predictor variables in larval growth and larval habitat suitability models.

Supplementary Table S1: Values for 50 experimental treatments averaged over the duration of each cup's involvement in the experiment (3-17 days) with ± SD for each value.

Cup Label	Temperature (°C)	Salinity (PSU)	pH	pCO_2 (ppm)	Ω Ar	DIC (µmol / kg)
A1	14.53 ± 1.7	27.27 ± 0.7	8.06 ± 0.01	362.51 ± 11.05	1.91 ± 0.06	1881.58 ± 27.2
A2	13.67 ± 0.88	37.98 ± 0.29	7.87 ± 0.05	645.38 ± 27.9	1.87 ± 0.38	2281.12 ± 37.98
A3	13.05 ± 0.35	24.63 ± 0.26	7.81 ± 0.11	667.54 ± 192.74	1 ± 0.2	1869.27 ± 24.63
A4	13.8 ± 1.56	32.94 ± 0.33	7.71 ± 0.06	978.49 ± 153.42	1.18 ± 0.13	2273.43 ± 32.94
A5	13.4 ± 0.36	30.27 ± 0.06	8.04 ± 0.03	391.84 ± 32.87	1.98 ± 0.11	1981.31 ± 30.27
B1	14.12 ± 0.77	18.66 ± 0.1	7.66 ± 0.04	818.34 ± 64.24	0.52 ± 0.03	1550.27 ± 18.66
B2	14.22 ± 1.64	13.77 ± 2.21	7.72 ± 0.04	732.91 ± 66.45	0.5 ± 0.11	1506.84 ± 13.77
B3	14.15 ± 1.46	33.99 ± 3.41	7.87 ± 0.03	649.05 ± 29.25	1.7 ± 0.28	2213.54 ± 33.99
B4	13.35 ± 0.35	15.8 ± 0.39	7.72 ± 0.03	722.02 ± 26.1	0.53 ± 0.07	1539.42 ± 15.8
B5	13.67 ± 0.7	21.32 ± 0.03	7.98 ± 0.05	406.71 ± 55.29	1.23 ± 0.11	1676.14 ± 21.32
C1	18.58 ± 0.59	14.21 ± 2.24	7.59 ± 0.03	918.9 ± 84.17	0.4 ± 0.09	1355.29 ± 14.21
C2	18.27 ± 0.15	15.81 ± 0.08	7.9 ± 0.05	471.32 ± 68.34	0.95 ± 0.08	1484.76 ± 15.81
C3	18.6 ± 0.69	35.83 ± 0.57	8.08 ± 0.02	363.77 ± 19.37	3.07 ± 0.3	2082.06 ± 35.83
C4	17.94 ± 1.91	21.64 ± 0.17	7.64 ± 0.04	926.82 ± 88.65	0.7 ± 0.06	1683.15 ± 21.65
C5	18.38 ± 1.72	26.93 ± 0.23	8.02 ± 0.02	385.4 ± 26.09	2 ± 0.12	1805.93 ± 26.93
D1	18.98 ± 0.96	38.62 ± 0.2	7.73 ± 0.06	1038.91 ± 135.38	1.86 ± 0.25	2530.58 ± 38.62
D2	18.15 ± 0.07	34.03 ± 0.15	7.77 ± 0.02	850.67 ± 70.7	1.63 ± 0.01	2270.58 ± 34.03
D3	19 ± 0.89	18.76 ± 0.11	7.74 ± 0.03	715.11 ± 42.53	0.78 ± 0.05	1571.28 ± 18.76
D4	18.55 ± 1.55	24.12 ± 0.42	7.66 ± 0.03	964.79 ± 79.56	0.88 ± 0.08	1865.15 ± 24.12
D5	19.04 ± 0.93	29.9 ± 0.45	7.71 ± 0.02	886.53 ± 42.44	1.22 ± 0.05	2008.19 ± 29.9
E1	20.77 ± 1.15	29.83 ± 0.58	8.05 ± 0.02	370.51 ± 22.76	2.54 ± 0.1	1876.01 ± 29.93
E2	21.65 ± 0.24	33.22 ± 0.53	7.68 ± 0.03	1078.68 ± 83.8	1.49 ± 0.09	2260.58 ± 33.22
E3	21.03 ± 0.9	27.11 ± 0.45	7.63 ± 0.03	1083.7 ± 73.06	1 ± 0.09	1949.39 ± 27.2
E4	21.57 ± 0.25	24.92 ± 0.06	7.7 ± 0.01	893.76 ± 19.41	1.1 ± 0.03	1872.38 ± 24.92
E5	21.25 ± 0.79	21.72 ± 0.25	7.64 ± 0.06	1026.1 ± 134.63	0.85 ± 0.09	1806.31 ± 21.72
F1	21.63 ± 0.25	39.02 ± 0.18	7.8 ± 0.05	887.29 ± 75	2.37 ± 0.32	2516.56 ± 39.02
F2	21.6 ± NA	12.93 ± NA	7.7 ± NA	737.68 ± NA	0.56 ± NA	1349.46 ± 12.93
F3	21.09 ± 1	18.44 ± 0.3	7.98 ± 0.02	406.76 ± 25.1	1.43 ± 0.14	1553.74 ± 18.52
F4	21.62 ± 0.27	36.34 ± 0.33	7.86 ± 0.04	690.39 ± 28.77	2.32 ± 0.31	2249.3 ± 36.34
F5	21.6 ± 0.2	15.81 ± 0.34	7.68 ± 0.04	872.8 ± 86.13	0.71 ± 0.07	1601.65 ± 15.81
G1	26.23 ± 0.72	13.29 ± 0.25	7.65 ± 0.01	847.26 ± 41.14	0.63 ± 0.03	1370.82 ± 13.29
G2	26.52 ± 0.52	21.04 ± 1.42	7.74 ± 0.02	738.53 ± 38.22	1.2 ± 0.13	1629.59 ± 21.04
G3	25.7 ± 1.39	19.05 ± 0.59	7.99 ± 0.04	397.33 ± 57.34	1.82 ± 0.15	1558.96 ± 19.05
G4	26.57 ± 0.58	33.62 ± 0.33	7.75 ± 0.04	895.3 ± 79.04	2.02 ± 0.12	2159.81 ± 33.62
G5	26.6 ± 0.61	30.52 ± 0.06	7.91 ± 0.12	563.93 ± 181.29	2.48 ± 0.55	1929.4 ± 30.52
H1	26.25 ± 0.73	16.07 ± 0.13	7.64 ± 0.04	991.17 ± 75.4	0.79 ± 0.08	1600.56 ± 16.07
H2	26.48 ± 0.55	28.43 ± 0.31	7.69 ± 0.03	957.28 ± 67.96	1.49 ± 0.09	1966.96 ± 28.43
H3	26.26 ± 0.57	24.97 ± 0.23	7.78 ± 0.03	734.55 ± 55.48	1.54 ± 0.09	1808.31 ± 24.97
H4	26.25 ± 0.87	39.9 ± 0.56	7.69 ± 0.05	1140.5 ± 95.24	2.29 ± 0.42	2508.28 ± 39.9
H5	26.55 ± 0.48	36.97 ± 0.36	7.66 ± 0.03	1205.3 ± 26.04	1.93 ± 0.2	2391.48 ± 36.97
I1	28.98 ± 1.33	19.31 ± 0.51	7.72 ± 0.05	817.17 ± 116.31	1.23 ± 0.07	1654.23 ± 19.31
I2	29.45 ± 0.76	31.3 ± 0.26	7.76 ± 0.05	800.5 ± 113.17	2.07 ± 0.17	1970.09 ± 31.3
I3	29.2 ± 0.83	34.08 ± 0.78	7.71 ± 0.08	992.82 ± 210.96	2.07 ± 0.25	2145.07 ± 34.08
I4	29.65 ± 0.21	13.63 ± 0.28	7.88 ± 0.02	533.45 ± 33.63	1.28 ± 0	1433.86 ± 13.63
I5	29.38 ± 0.61	25.1 ± 0.19	7.63 ± 0.01	1123.29 ± 46	1.33 ± 0.05	1920.18 ± 25.1
J1	28.88 ± 0.9	28.62 ± 0.69	7.7 ± 0.01	927.55 ± 35.11	1.67 ± 0.05	1946.18 ± 28.62
J2	28.42 ± 1.22	22.36 ± 0.37	7.99 ± 0.05	411.59 ± 56.5	2.28 ± 0.15	1635.99 ± 22.36
J3	28.96 ± 0.79	34.98 ± 4.14	7.82 ± 0.06	730.55 ± 83.26	2.61 ± 0.6	2100.77 ± 34.98
J4	28.85 ± 0.93	39.5 ± 0.79	7.75 ± 0.03	929.75 ± 31.98	2.68 ± 0.38	2357.61 ± 39.5
J5	28.8 ± 1.24	16.41 ± 0.42	7.93 ± 0.01	460.82 ± 31.8	1.57 ± 0.06	1473.59 ± 16.41

Supplementary Table S2: Model selection process for larval growth Generalized Additive Model. Larval growth rate (Gr) is predicted using temperature (Temp), salinity (Sal), pH, pCO_2, and aragonite saturation (Ar) using thin plate regression splines for individual variables (s()) and full tensor product splines for interacting variables (te()), both using REML smoothing. The final model is highlighted in dark grey, with the 5 next best performing models in light grey.

	Formula	df	logLik	AICc	AICc Rank	BIC	BIC Rank	R^2
Null	Gr ~ 1	1	-146.68	297.62	43	301.19	42	0
One Predictor	Gr ~ s(Temp)	2	-134.43	275.37	28	280.59	27	0.37
	Gr ~ s(Sal)	3.41	-135.81	282.98	39	291.17	38	0.32
	Gr ~ s(pH)	2	-146.32	299.17	44	304.38	44	-0.01
	Gr ~ s(pCO2)	2.39	-143.49	295.1	42	301.38	43	0.09
	Gr ~ s(Ar)	2.61	-138.09	285.09	41	291.86	39	0.27
Two Predictors	Gr ~ s(Temp) + s(Sal)	5.66	-111.71	242.17	14	253.68	15	0.73
	Gr ~ s(Temp) + s(pH)	3	-134.42	277.74	31	284.5	30	0.36
	Gr ~ s(Temp) + s(pCO2)	3.19	-133	275.75	29	283.04	28	0.39
	Gr ~ s(Temp) + s(Ar)	3.22	-129.13	268.17	25	275.54	25	0.48
	Gr ~ s(Sal) + s(pH)	4.58	-133.51	281.33	36	291.03	37	0.36
	Gr ~ s(Sal) + s(pCO2)	4.63	-132.85	280.15	35	289.9	35	0.38
Three Predictors	Gr ~ s(Temp) + s(Sal) + s(pH)	6.29	-110.63	239.89	11	251.36	13	0.73
	Gr ~ s(Temp) + s(Sal) + s(pCO2)	6.28	-110.82	240.25	12	251.72	14	0.73
Add Interactions	Gr ~ te(Temp, Sal)	18.95	-72.34	221.18	1	227.76	1	0.92
	Gr ~ te(Temp, Sal) + s(pH)	19.75	-72.61	227.35	6	231.66	3	0.92
	Gr ~ te(Temp, Sal) + s(pCO2)	19.74	-72.62	227.31	5	231.65	2	0.92
	Gr ~ te(Temp, pH)	4	-134.06	279.48	34	287.68	33	0.36
	Gr ~ te(Temp, pH) + s(Sal)	7.33	-109.8	241.31	13	253.79	16	0.74
	Gr ~ te(Temp, pCO2)	4	-132.23	275.82	30	284.01	29	0.4
	Gr ~ te(Temp, pCO2) + s(Sal)	7.26	-110.82	243.13	15	255.55	18	0.73
	Gr ~ te(Temp, Ar)	8.08	-120.73	270.96	26	285.13	32	0.59
	Gr ~ te(Sal, pH)	6.13	-131.63	282.84	38	294.64	40	0.39
	Gr ~ te(Sal, pH) + s(Temp)	8.06	-110.36	245.61	18	258.93	22	0.73
	Gr ~ te(Sal, pCO2)	6.39	-131.48	283.01	40	294.98	41	0.39
	Gr ~ te(Sal, pCO2) + s(Temp)	8.13	-110.45	245.65	19	258.93	22	0.73
Interactions Smooth by Linear	Gr ~ te(Sal, by = Temp)	5.25	-103.53	222.41	2	232.57	4	0.81
	Gr ~ te(Sal, by = Temp) + s(pH)	6.27	-102.59	223.33	3	234.63	5	0.81
	Gr ~ te(Sal, by = Temp) + s(pCO2)	6.27	-102.77	223.71	4	235.01	6	0.81
	Gr ~ te(Temp, by = Sal)	3	-119.62	248.13	21	254.89	17	0.65
	Gr ~ te(Temp, by = Sal) + s(pH)	4	-119.61	250.59	22	258.78	20	0.64
	Gr ~ te(Temp, by = Sal) + s(pCO2)	4	-119.61	250.59	22	258.79	21	0.64
	Gr ~ te(Temp, by = pH)	3	-134.5	277.89	32	284.65	31	0.36
	Gr ~ te(Temp, by = pH) + s(Sal)	6.3	-110.47	239.65	10	251.15	12	0.74
	Gr ~ te(Temp, by = pCO2)	3	-132.25	273.39	27	280.15	26	0.41
	Gr ~ te(Temp, by = pCO2) + s(Sal)	6.98	-112.09	247.16	20	260	23	0.71
	Gr ~ te(Temp, by = Ar)	4.48	-124.93	263.76	24	273.25	24	0.55
	Gr ~ te(Sal, by = pH)	4.46	-133.74	281.37	37	290.86	36	0.36
	Gr ~ te(Sal, by = pH) + s(Temp)	6.01	-110.77	239.18	9	250.28	10	0.74
	Gr ~ te(Sal, by = pCO2)	4.57	-132.07	278.24	33	287.83	34	0.4
	Gr ~ te(Sal, by = pCO2) + s(Temp)	7.9	-109.58	244.71	16	258.17	19	0.74
Two Interactions	Gr ~ te(Temp, pH) + te(Temp, Sal)	20.3	-78.41	252.24	23	250.42	11	0.89
	Gr ~ te(Temp, pCO2) + te(Temp, Sal)	17.8	-82.92	235.9	7	244.81	8	0.88
	Gr ~ te(Sal, pH) + te(Temp, Sal)	18.83	-75.07	239.13	8	240.41	7	0.91
	Gr ~ te(Sal, pCO2) + te(Temp, Sal)	18.91	-77.55	245.5	17	246.11	9	0.9

Supplementary Table S3: Model selection process for larval habitat suitability GLM. Suitability was modeled with a multiple logistic regression using temperature (Temp), salinity (Sal), pH, pCO_2, and aragonite saturation (Ar) as predictor variables. Model comparisons used AICc, BIC, and McFadden's Adjusted Pseudo R^2. The final model is highlighted in dark grey, with the 5 preceding (by AICc) in light grey.

	Formula	AICc	AICc Rank	BIC	BIC Rank	Pseudo R^2
Null	Suitability ~ NULL	64.77	48	66.6	42	-0.03
One Predictor	Suitability ~ Temp	55.49	20	59.06	15	0.12
	Suitability ~ Sal	62.26	42	65.83	38	0.01
	Suitability ~ pH	65.74	49	69.31	47	-0.04
	Suitability ~ pCO2	62.83	45	66.4	41	0
	Suitability ~ Ar	62.63	44	66.2	39	0
Two Predictors	Suitability ~ Temp + Sal	52.66	14	57.88	14	0.17
	Suitability ~ Temp + pH	57.25	32	62.46	23	0.1
	Suitability ~ Temp + pCO2	55.57	21	60.78	16	0.12
	Suitability ~ Temp + Ar	56.37	27	61.59	21	0.11
	Suitability ~ Sal + pH	62.59	43	67.81	44	0.01
	Suitability ~ Sal + pCO2	62.15	41	67.36	43	0.02
Three Predictors	Suitability ~ Temp + Sal + pH	54.46	18	61.22	20	0.15
	Suitability ~ Temp + Sal + pCO2	54.45	17	61.21	19	0.15
One Quadratic Term	Suitability ~ Temp + Temp^2	56.97	31	62.18	22	0.1
	Suitability ~ Sal + Sal^2	44.89	13	50.11	13	0.29
	Suitability ~ Temp + Temp^2 + Sal	54.3	16	61.06	18	0.15
	Suitability ~ Temp + Temp^2 + pH	58.98	39	65.73	37	0.07
	Suitability ~ Temp + Temp^2 + pCO2	57.52	34	64.28	28	0.1
	Suitability ~ Temp + Temp^2 + Ar	57.84	35	64.59	33	0.09
	Suitability ~ Sal + Sal^2 + pH	41.95	12	48.71	12	0.34
	Suitability ~ Sal + Sal^2 + pCO2	41.12	11	47.88	11	0.36
	Suitability ~ Sal + Sal^2 + Temp	21.77	1	28.53	1	0.67
Two Quadratic Terms	Suitability ~ Temp + Temp^2 + Sal + pH	56.36	26	64.56	31	0.12
	Suitability ~ Temp + Temp^2 + Sal + pCO2	56.39	28	64.58	32	0.12
	Suitability ~ Sal + Sal^2 + Temp + pH	22.35	3	30.55	3	0.67
	Suitability ~ Sal + Sal^2 + Temp + pCO2	21.88	2	30.07	2	0.67
	Suitability ~ Sal + Sal^2 + Temp + Temp^2	24.24	7	32.44	5	0.64
	Suitability ~ Sal + Sal^2 + Temp + Temp^2 + pH	24.65	9	34.17	9	0.64
	Suitability ~ Sal + Sal^2 + Temp + Temp^2 + pCO2	24.16	6	33.68	7	0.65
Interactions	Suitability ~ Temp + Sal + Temp * Sal	54.12	15	60.88	17	0.15
	Suitability ~ Temp + Sal + Temp * Sal + pH	56.33	25	64.53	30	0.12
	Suitability ~ Temp + Sal + Temp * Sal + pCO2	56.29	24	64.48	29	0.12
	Suitability ~ Temp + pH + Temp * pH	59.56	40	66.32	40	0.06
	Suitability ~ Temp + pH + Temp * pH + Sal	56.06	23	64.26	27	0.13
	Suitability ~ Temp + pCO2 + Temp * pCO2	57.85	36	64.61	34	0.09
	Suitability ~ Temp + pCO2 + Temp * pCO2 + Sal	56.45	29	64.64	35	0.12
	Suitability ~ Temp + Ar + Temp * Ar	57.49	33	64.25	26	0.1
	Suitability ~ Sal + pH + Sal * pH	63.44	46	70.2	48	0
	Suitability ~ Sal + pH + Sal * pH + Temp	56.89	30	65.09	36	0.11
	Suitability ~ Sal + pCO2 + Sal * pCO2	64.2	47	70.96	49	-0.01
	Suitability ~ Sal + pCO2 + Sal * pCO2 + Temp	55.02	19	63.22	24	0.14
Quadratic with Interactions	Suitability ~ Temp + Sal + Temp * Sal + Temp^2	56.01	22	64.2	25	0.13
	Suitability ~ Temp + Sal + Temp * Sal + Sal^2	23.5	4	31.7	4	0.65
	Suitability ~ Temp + Sal + Temp * Sal + pH + Temp^2	58.41	38	67.93	46	0.1
	Suitability ~ Temp + Sal + Temp * Sal + pH + Sal^2	24.51	8	34.03	8	0.64
	Suitability ~ Temp + Sal + Temp * Sal + pCO2 + Temp^2	58.39	37	67.91	45	0.1
	Suitability ~ Temp + Sal + Temp * Sal + pCO2 + Sal^2	24.15	5	33.67	6	0.65
	Suitability ~ Temp + Temp^2 + Sal + Sal^2 + Temp * Sal	26.09	10	35.61	10	0.61

Supplementary Table S4: Case study field data from two restoration sites in Washington State between the years 2014 and 2019. Field data are listed on the left, and modeled response variables on the right of the vertical line. Values for °C and PSU are depth-averaged means ± sd.

Site	Year	°C	PSU	Dates	Sample Frequency	Instrument	Coordinates	Growth Rate (µm/day)	PLD (days)	Habitat Suitability Likelihood %
Liberty Bay	2017	16.44 ± 0.59	29.09 ± 0.58	Jul. 7-Aug. 23	Twice weekly through water column	Pro Plus YSI	47.734°N 122.648°W	4.88	21.3	31.12
Liberty Bay	2018	17.36 ± 1.15	29.58 ± 2.43	Jul. 7-Aug. 23	Twice weekly through water column	Pro Plus YSI	47.734°N 122.648°W	5.59	18.6	49.9
Liberty Bay	2019	16.71 ± 1.07	27.7 ± 0.81	Jul. 7-Aug 23	Twice weekly through water column	Pro Plus YSI	47.734°N 122.648°W	5.12	20.3	26.65
Fidalgo Bay	2017	16.6 ± 1.3	29 ± NA	Jul. 11-14	11 times daily through water column	Hatch DS5 Multiprobe	48.4823°N 122.580°W	5.01	20.7	33.66
Fidalgo Bay	2014	16.5 ± 0.5	28.4 ± 0.25	Jul. 2, 14	Once daily through water column	Hatch DS5 Multiprobe	48.4789°N 122.579°W	4.94	21.0	28.8

Supplementary Table S5: Mean temperature and salinity from the Salish Sea Hydrodynamic Model from July 5- August 23, 2014 and 2095 in the 19 state-managed Olympia oyster restoration sites in Washington State with average model-predicted PLD in each site for each year. The final two columns show change in projected PLD in days and as percentages between 2014 and 2095. White rows were those where site temperatures were outside the limits of our GAM (<13°C), so were removed.

	2014			2095			Δ PLD	
Site	Temp.	Sal.	PLD	Temp.	Sal.	PLD	days	%
Bellingham Bay	13.6	26.5	41.6	16.5	25.9	21.7	-19.9	-47.8
Budd Inlet	15	25.3	29.2	16.5	26	21.5	-7.7	-26.4
Discovery Bay	10.8	31	--	14.8	28.8	--	--	--
Drayton Harbor	11.3	28.8	--	14.2	28.3	--	--	--
Dyes Inlet	13.5	28.8	44.5	16.9	28.4	19.7	-24.8	-55.7
Fidalgo Bay	12.7	28	--	16.2	26.4	--	--	--
Henderson Bay	14	27.8	37.5	16.6	27.7	20.6	-16.9	-45.1
Kilisut Harbor	10.9	30.5	--	14.4	29	--	--	--
Liberty Bay	12.5	28.8	--	15.9	28.5	--	--	--
Padilla Bay	12.6	27.7	--	16.6	26	--	--	--
Port Gamble Bay	12.3	30.2	--	15.2	29	--	--	--
Port Orchard Pass	13	28.8	53.2	15.9	28.3	23.2	-30	-56.4
Quilcene Bay	10.9	29.8	--	13.7	28.9	--	--	--
Samish Bay	13.1	27.9	50.6	17.5	26.2	18.5	-32.1	-63.4
Sequim Bay	11.2	30.5	--	15.7	28	--	--	--
Similk Bay	12.1	26.8	--	14.7	27.1	--	--	--
Sinclair Inlet	13.4	28.6	45.5	16.3	28.4	21.7	-23.8	-52.3
Squaxin Island	13.5	28.6	44.1	15.9	28.1	23.2	-20.9	-47.4
Union River	15.4	28.1	25.8	19.4	27.8	14.2	-11.6	-45
